# What Denture Service Costs Dentists*This is the first of a series of articles under this title which will seek to present what has been learned about the cost of denture construction by a study of reports sent in by dentists. The second article is expected to appear in the February issue.—Editor, *Digest*. Reprinted and compiled from *Dental Digest*, January issue.

**Published:** 1922-02

**Authors:** George Wood Clapp

**Affiliations:** New York


### WHAT DENTURE SERVICE COSTS DENTISTS*

BY GEORGE WOOD CLAPP, D.D.S., NEW YORK

Reports from 38 practices, all with annual gross receipts of less than $5,000 per year, seem to show that these dentists require, on the average, 5 hours of chair-time for each set of full maxillary and mandibular dentures for one month, at an average cost to the practices of about $20.

By "chair-time" is meant the time required to interview the patient, arrange for the service, examine the mouth, take the impressions, register correct occlusion, select the form, size and color of the teeth and make subsequent minor adjustments.

Reports from 76 practices, with annual gross receipts ranging from $5,000 to $10,000, seem to show that these dentists require, on the average, 5¾ hours of chair-time for similar dentures, with an average cost to the practices of a little more than $41.

Reports from 17 practices, with annual gross receipts ranging from $10,000 to $15,000, seem to show that these dentists require, on the average, 6 hours of chair-time for the making of such dentures, with an average cost to the practices of about $72. These 17 dentists do their own

* This is the first of a series of articles under this title which will seek to present what has been learned about the cost of denture construction by a study of reports sent in by dentists. The second article is expected to appear in the February issue.—EDITOR, *Digest*. Reprinted and compiled from *Dental Digest*, January issue.

laboratory work with an average expenditure of $7\frac{1}{2}$ hours' time, which, on the average, costs the practices about $87. This makes the average total cost of the dentures $159.

Reports from 8 practices, with annual gross receipts ranging from $15,000 to $20,000, seem to show that these dentists require, on the average, 5 hours of chair-time for the dentures, at an average cost to the practices of about $86. In 6 practices of this group the average total labor cost for the dentures appears to be about $115.

Reports from 9 practices, with annual gross receipts ranging from $20,000 to $57,000, vary so greatly that averages are of no practical value. It may be interesting to know that the cost of dentures in this class appears to range from $107 to $703, with an average of about $226.

### IT IS UNSAFE TO TRUST AVERAGES

A dentist who has an inadequate accounting system might decide, "My practice shows annual gross receipts closely corresponding to one of the averages given. If the average cost to such a practice for the chair-time required to make full maxillary and mandibular dentures is $41, my cost is probably not less than that amount and my minimum fee should be at least that amount plus the laboratory charge." Such a conclusion will undoubtedly save him from a loss which would result from a lower fee, but it has no other value, and it might not protect him from actual loss if his production costs were higher than those given. Perhaps the greatest value of such an average cost is that it should be enough higher than a dentist's conception of his own costs to start him upon some plan for ascertaining his exact costs.

### FACTORS WHICH MAKE THE AVERAGE UNSAFE GUIDES

The real value of these averages is much less than the apparent value because they are made from practices which differ greatly from one another. Thus, the average cost, when the average annual gross receipts are $7,100

per year, and the average annual expenses $2,600 per year, is obtained by averaging reports from 76 practices in which the annual gross receipts range from $5,000 to $10,000, and the annual expenses from a little over $1,000 to a little over $5,900. As a matter of fact, only 7 practices in the group had annual gross receipts of exactly $7,100.

Another factor which lessens the value of these averages as guides in the individual practice is that the figures here given are based upon the earnings or the annual gross receipts in 1,000 income hours per year, when, in fact, the dentist may have had many more income hours in the year, perhaps as many as 2,000. If the dentist had 2,000 income hours per year, his costs would be only one-half of those estimated upon the basis of 1,000 income hours.

The basis for the 1,000 income hours has been frequently given, but can not be too often repeated until it is generally understood. It is as follows:

Professional work should be in that proportion to recreation which will conserve the vitality, and in that proportion to study which will permit continued professional development. Unusual individuals have been able to work all day and half the night continuously for years without physical breakdown, but the best evidence available seems to show that the dentist will live longer and do more work and better work if he spends not more than 2,000 office hours per year.

In the present deplorable state of knowledge concerning economic administration of dental practice, that dentist will do well who, out of 2,000 office hours, averages 1,000 income hours for each of the 20 years between the ages of 35 and 55. The fact that with greater knowledge of economic administration the average number of income hours can be greatly increased, without increasing the office hours, with great profit to all concerned, is, as Kipling says, "another story."

The costs based upon the earnings of the reported annual gross receipts in 1,000 income hours per year may have one great value. They are probably close approximations to what the costs would be if the practices were conducted in such way that the dentist worked 1,000 income hours and had sufficient time for home life, recreation and professional study. They are probably more important from this particular point of view than as exact reports of costs in these practices, under present conditions.

It is expected that a more detailed study of costs in Class I practices will appear in the next issue.

Reports have been received from 600 practices located in towns and cities of various sizes, throughout the country.

Class I. Reports from 176 practices, all with gross annual receipts of less than $5,000 annually. Average annual gross receipts, $3,756. Average net receipts available for remuneration, $2,463. These practices constitute practically 30 per cent. of the entire number reporting.

Class II. Reports from 310 practices, all with gross annual receipts from $5,000 to $10,000. Average annual gross receipts, $7,000. Average annual net receipts available for remuneration, $4,500. These practices constitute about 50 per cent. of the entire number reporting.

Class III. Reports from 82 practices with annual gross receipts from $10,000 to $15,000 per year. Average annual gross receipts, $12,110. Average annual net receipts available for remuneration, $7,134.

Class IV. Reports from 20 practices, all with annual gross receipts between $15,000 and $20,000 per year. Average annual gross receipts, $16,949. Average annual net receipts, available for remuneration, $10,988.

Class V. Reports from 12 practices all with annual gross receipts of more than $20,000 annually. The receipts in this group and the conditions of conduct of the practice vary so widely that averages are of no value, though it may be interesting to know that the average amount available for remuneration is apparently $15,737.

The dentists in Classes III, IV and V constitute practically 20 per cent. of the total number reporting.

